# Test Item Taxonomy Based on Functional Criteria

**DOI:** 10.3389/fpsyg.2018.01175

**Published:** 2018-07-10

**Authors:** Rafael Moreno, Rafael J. Martínez, José Muñiz

**Affiliations:** ^1^Department of Experimental Psychology, University of Seville, Seville, Spain; ^2^Department of Psychology, University of Oviedo, Oviedo, Spain

**Keywords:** items, tests, taxonomy, item formats, classification, generation

## Abstract

There are many taxonomies that try to classify and apply some consistency to the very many item types currently in existence. They all have various limitations, however, such as ambiguous classification criteria, little discrimination between format types, and referring almost exclusively to pen-and-paper or screen-based items. This paper aims to overcome these limitations by proposing a new item format taxonomy based on functional criteria. Current classifications are reviewed, the criteria they are based on are examined and their limitations are identified. The proposed alternative classification identifies four essential components of items according to function: the structure of the included content, the device used for transmission of the question to the examinee, the device for receiving the response, and the instructions to the examinee about how to understand and respond to the item. The combination of different facets of these four components allows any format of item to be classified, both existing formats and those that may appear in the future. In addition to systematically and coherently classifying items, this new taxonomy may also be of great utility in the construction and research of new items. The proposed model is illustrated by examples showing how specific items are classified, using a checklist as a guide.

## Introduction

Test items are the basic units, the building blocks of psychological and educational testing, of which there are a huge variety. Many different classification systems have been put forward in an attempt to give that variety some structure. Some classify items in terms of their content, specifically in terms of skills being evaluated (Magno, 2009), or the levels of cognitive function that the constructs being measured require (Osterlind, 1998); others add criteria about format or the way content is presented (Osterlind and Merz, 1994; Rauthmann, 2011), such as the codes of expression of item components, their spatial distribution, or the medium through which they are presented. Many classifications refer exclusively to formats, which is the focus of this work. Haladyna and Rodriguez (2013) expanded the distinction between selected-response and constructed-response formats, also adopted by Osterlind (1998), Downing (2006), Sireci and Zenisky (2006, 2016) and Rodriguez (2016). Refining that distinction, Scalise and Gifford (2006) established seven types, from fully selected to fully constructed, looking at the task required by the items: multiple-choice, selection, reordering, substitution, completion, construction, and presentation. These types allow the evaluation of more complex skills than those that are usually encouraged by the ubiquitous Multiple Choice Items. The evaluation of these more complex skills is facilitated by including diverse media such as sound, animation or video; using different answer modes such as dragging objects on screens, constructing or modifying graphics, or clicking in areas of an image; and also by creating a flow of successive linked items. However, these classification criteria refer to ambiguous definitions of item components. Sometimes they are in morphological terms, with the *stem* or *lead-in* as the initial part of the item, and the *response options* as what follows in a list. Others are given in terms of function, with the stems framing the evaluated content, which is completed by the options. This ambiguity can lead to inconsistencies (e.g., Moreno et al., 2015). There are stems which give instructions, such as “Choose the correct option from the following…", where the evaluated content appears in what are, morphologically, options. There is also content in the form of options, as usual in *Multiple-Choice* items. There are also apparent options which are not, in fact, functional options. This happens in *Ranking and sequencing* formats, as the true options are the possible orders from which a correct order must be chosen; also in *Multiple-Answer* and *Select-All-That-Apply*, as the real options are not those presented, but rather the possible combinations. Similarly, there are options that do not follow the usual appearances, for example the *Sore-finger* format, where words in a text must be marked if they are thought to be correct.

From an epistemological point of view, the usual classification criteria have been arrived at inductively without theoretical foundation. Another notable limitation is that there is little to differentiate the variety of formats included in each category. The twenty one constructed-response formats collected by Haladyna and Rodriguez (2013) are presented alphabetically; and within the selected-response formats the different types of *Multiple-choice*, *True-False*, and *Matching* are listed but not classified. The seven types proposed by Scalise and Gifford (2006) are more structured, but missing the formats included in each type. The gaps in classification will only increase in future with the appearance of new formats thanks to the influence of new technologies in testing (Drasgow, 2016). In addition, classification criteria are applied in a limited manner. The taxonomy from Rodriguez (2016) only refers to selected-response items, and Osterlind and Merz (1994) pay “decidedly more attention (…) to organising constructed-response formats,” mentioning selected-type formats “for reasons of completeness and comparison rather than as any deliberate attempt to organise them” (p. 135). Taxonomies from Scalise and Gifford (2006) and Sireci and Zenisky (2006, 2016) are focused on computer-based assessment formats. In contrast, Haladyna and Rodriguez (2013) try to classify all of the above item types, gathering together almost forty different formats, and adding those collected by Sireci and Zenisky (2006). Nevertheless, as with other classification attempts, and with the exception of *Interview* and *Oral Examination*, they are concerned with items that are predominantly on paper or on screen, and they omit other item types such as tactile, which are fundamental when evaluating blind people, or olfactory and taste testing, which are essential in some neuropsychological or perception research.

Within this context, this research aims to offer a taxonomic model of test item formats, based on universal, rigorous, functional criteria, which will overcome the limitations of current classification systems. In addition to classifying existing and emerging items, the new taxonomy can guide the construction of new items. For that reason, the proposed taxonomy may be very useful to researchers and professionals who develop evaluation instruments in health or social sciences, psychology, and education. The universal and precise nature of the taxonomy may also be a great help in generating items which are invariant between groups and cultures (Byrne and van de Vijver, 2017; Gómez-Benito et al., 2017). If the criteria on which the taxonomy is based are universal and therefore intercultural invariants, the classifications of the items they allow will also be. This does not eliminate the need for the corresponding adaptations of the items (Muñiz et al., 2013; International Test Commission, 2017), but it adds the advantage of carrying them out within a taxonomic model based on functional criteria.

## Components of the Proposed Taxonomic Model

Haladyna and Rodriguez (2013, p. 3) stated that an “item is a device for obtaining information about a test taker’s domain of knowledge and skills or a domain that define a construct”. An item fulfils this function in so far as it allows effective, two-way communication between the evaluator and the test-taker or examinee, allowing the evaluator to ensure that the examinee understands what is being asked of them and then collect their response. This requires a configuration of components which give the items stability and reproducibility, and which distinguish them as much as possible from informal questions such as those found in everyday conversation. In the taxonomic model of formats we propose here there are four essential components which define each type of item. The components are: the *content structure* of the item, a *transmission device* of information to the examinee, a *reception device* for their response, and an *instruction device*. Any item is defined when these four components of the model are specified and their functions understood. Item *content structure* is the central component as it gives the rest meaning. It does not refer to an item’s particular knowledge, skills or constructs, but rather to the organisation of item content, focussing on the object of study about which information is sought within a contextual framework, according to some subject domain. It is analogous to the semantic structure of natural language highlighted by research on Knowledge Representation, (e.g., Helbig, 2006), used in Expert Systems and Ontology Engineering in a way that is transferrable to different fields of application. The evaluator attempts to gather information via this content structure, which involves reducing the uncertainty between the target information and other alternatives (Gallager, 1968). The three remaining components are the vehicles by which this content structure is communicated. They are differentiated as follows, based on Communication Theory (Shannon, 1948; Anderson, 1996): The *transmission device* is that part of the content structure presented to the examinee which they are asked to respond to. The *reception device* allows the expression of the examinee’s response to be collected, for example via boxes or screens where the examinee writes text or makes marks. Finally, the *instruction device* provides indications about the transmission and reception devices, and other aspects such as examinee identification data, or the marks awarded for each answer. The information provided by instructions is different to the information in the transmission device. The latter refers to the item content whereas the instructions give information about the transmission and reception devices. For example, in the Short-Answer item “What is the capital of Italy?” the word “What” and the question mark together make up the implicit expression of the instruction indicating what is being requested. Explicit expression would be used when essential instruction about how or where to respond to the question may not be obvious, which would be done by indicating “Answer the following question, writing your answer in the space below.”

The four components are defined operationally by means of the various possibilities or cases of their *facets*, allowing a detailed classification of items.

## Facets of Content Structure

There are three facets to this component: domain, frame, and object of study.

### Domain

This is the area or field which includes the other two facets and gives them meaning. It could be wide or narrow, for example a particular discipline, or a specific subject within a discipline. The possibilities of this facet are specified by the *number of domains* in the item, a single domain being common in studies on a single topic, multiple domains in multidisciplinary items, and none in projective tests.

### Frame

Every domain is composed of a set of related contents. In each item some receive the central attention as objects of study, leaving the rest as a frame or context. Therefore, the frame can be composed of people, institutions, objects, situations, moments, places, and any other aspect that functions as circumstances or setting. Since there can be many contents that can fulfil this function, in each item only those considered most relevant or convenient for the desired evaluation are specified. Thus, once again the *number of frames* transmitted in the item specifies the possibilities of this facet, one being the most usual, multiple in *Matching* formats, and none in some projective tests.

### Object of Study

This is the concept about which information is wanted in each item. It is made up of an attribute which may be simple or complex, and which can be assigned different values or cases. One or more of these are the *target values* which the examinee’s response is measured against, the others are *alternative values*. The function of the target values and their differentiation from the alternatives comes from the correspondence that only the targets have with the frame. For example, to gather information in the domain of political geography about which city is the capital of Italy, the object of study is “city,” “Rome” is the target value, and the others are alternatives for the frame “capital of Italy.” Because of the selective role that the frame plays in terms of targets, if we change the frame for the same object of study, we can also change the targets; so, for the frame “capital of Lombardy,” the target would be “Milan,” while “Rome” or any other city would be alternative values. The target-frame correspondence is publicly fixed in some domains, and in others it is established for each examinee according to their history and circumstances. The former is used to determine if the examinee’s response is “correct” in that domain when evaluating knowledge or skills, and the latter to determine what is “preferred” or “agreed” by each person, when evaluating opinions or attitudes.

**FIGURE 1:**
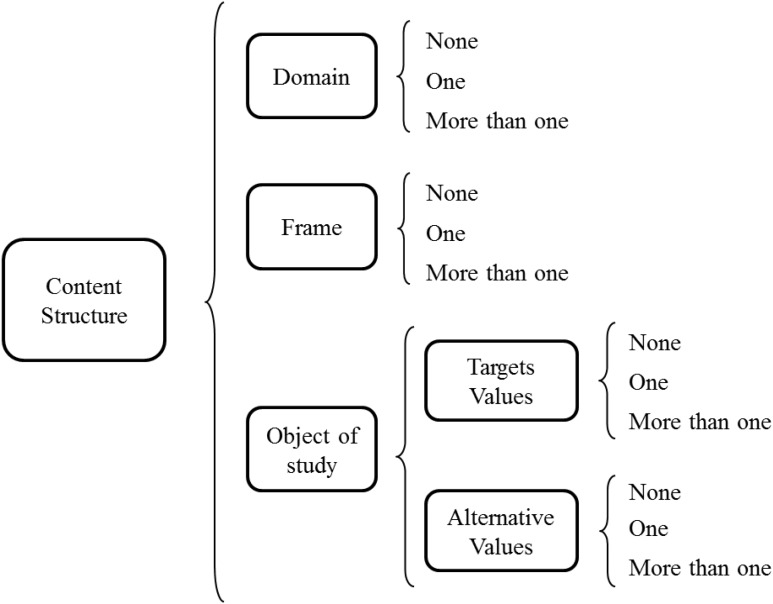
Possibilities of the Content Structure facets for each item.

The possibilities for each item’s object of study are the *number of target values and alternatives* in the transmission device. This covers the omission of both values, such as in constructed-response items like “What is the capital city of Italy?”. It also covers the inclusion of a single target or alternative value, such as in *True–False* formats when including a value that corresponds to a particular frame or not, such as “Rome is the capital of Italy” or “Milan is the capital of Italy” for the same object of study and frame as the previous example. Another possibility is to communicate a target together with one or more alternative values such as in the triad “Florence, Milan, Rome” for the aforementioned frame; formats such as *Alternative Choice*, *Bipolar Adjectives*, and *Two-option* formats include one alternative value along with the target, *Three-Option* includes one more, and there are four or more in *Extended Multiple-Choice*, *Uncued Multiple Choice*, and usual *Likert* items. These possibilities also encompass items with various target and alternative values, such as in *Multiple-Answer* and *Select-All-That-Apply* items. They also cover each of the objects of study in multiple items; for example*, Concept Map* and *Interview* do not have any target or alternative values, whereas *Matching and Multiple true-false* include varieties of both. **Figure 1** summarises the possible content structure along with possible combinations of the various facets of an item.

Each of these possibilities affects the type of response required. Although the request must always be for the examinee to communicate what they think is the target, that may happen in two ways, which correspond to the difference between constructed and selected-response items: the former when the transmission device includes neither targets nor alternatives, and the latter by indicating whether each value presented with the corresponding frame is thought to be the target or not.

## Device Facets

The three devices function according to the following facets which they necessarily present: sensory modality, code of expression, spatiotemporal configuration, and physical medium. These specify how each device functions, and they can be identified in the items’ morphological characteristics or specific materials.

### Sensory Modality

This refers to the channels that the evaluator and the examinee use. The possibilities of this facet may be characterised according to the five senses. To activate the examinee’s *visual* channel, the transmission device would usually consist of lines, text, or static or moving images such as in a *Video-Based Task*; the reception device would usually be spaces, lines, ovals, circles, boxes, or tables where the answers can be given by marking text, crosses, filling-in and other forms; in computers it is common to have figures to modify, areas to drag text or numbers to, and drop-down menus to click in. For the *olfactory, auditory, taste*, and *touch* modalities the transmission device consists of smells such as in olfactometers, sounds, tastes and tactile stimuli. Because the examinee’s responses may be in the same modalities, the reception device must be appropriate, such as audio or video recordings, or it must make use of the respective senses of the evaluators. The instructions can encompass all modalities, although it is almost always visual or auditory modalities that are used because of their versatility and ease of understanding. Nevertheless, in the same way that there are instructions which include test items such as visual models to imitate, evaluation of infants or those with certain deficits may include instigation via touch to follow a model being presented. Each device in an item may involve more than one modality, such as a path to follow which includes visual and auditory indicators, and even smells or tactile indicators or simulations with sound and images. Similarly, the three devices may present differing modalities within each item, such as an odour transmitter and a visual receptor using circles to mark responses, and all the combinations thereof.

### Code of Expression

This consists of the system of rules used in formulating the item. There are many possibilities for this facet, given that any system of rules that allows the function of each device to be expressed may conform to a code. For example, the signs in a visual transmission device include written text of natural languages, but also numerical, formulaic and symbolic writing such as in mathematics and logic, and figures and icons such as emoticons or traffic signals; some are indicated by the names of the formats such as *Single Numerical* and *Format Map*. Codes which may be expressed aurally include musical codes, Morse code, spoken languages; and smells are used in the evaluation of perfumers and wine-tasters, along with visual and taste codes. Tactile codes include Braille, and codes made up of the intensity of stimulation in biofeedback evaluation. Codes in reception devices can be identified in format names such as *Blankety-Blank* and *Grid-In*; other formats indicate response codes in the device, such as *Create-a-tree*, *Drag-and-Connect*, *Graphical Modelling*, and *Short Verbal Constructed-Response*; while others have both, such as *Fill-in-the-blank*, *Highlighting Text*, *Limited Figural Drawing*, *Matrix Completion*, and *On-Screen Drawing*. Different codes are also found in instructions; the use of both written and spoken natural languages is widespread. The wide variety of possibilities given here as examples may be grouped into two categories: *generic* codes used in items but developed elsewhere, such as Braille or written and spoken English; and *specific* codes for items, such as putting a cross only in the circle the examinee thinks is the target value, or dragging the target to a particular area of the screen. In the same way as with the sensory modalities, each device in an item may include a mix of two or more codes, of the same category or of the two categories, such as the transmission using text, numbers and figures in mathematics, or a particular code in a receptor specifying text to be completed in natural language. These examples also illustrate the fact that the codes do not need to be the same in the different item devices, as all combinations are accommodated.

### Spatiotemporal Configuration

This facet is the manner of spatiotemporal organisation for each device. The possibilities when considering both *space and time* are *integrated* as a unit, or *separate* elements. A device may therefore be integrated in both dimensions, such as a transmission consisting of a paragraph presented only once. It may be integrated in space, and separate in time, such as the transmission of successive information which is superimposed in one location. There may also be separation in space and integration in time, such as transmissions made up of testlets of different separate texts given simultaneously; such a spatial separation may be configured horizontally or vertically, such as in *Multiple-choice* or *Likert* formats, respectively, or irregularly like pieces of a puzzle. Separation in space and in time is also possible. Not only does each device have more than one possibility, the configurations of the three devices may differ from each other. For example, in one item, every device may be integrated, whereas in another item, two may be integrated and one separate.

All of the configuration possibilities for each device also occur for each one with respect to the others. So, for example, in *Multiple-choice* the target and alternative values transmitted separately on different lines usually appear integrated in space and time, with the reception device also spatially separated with circles to fill in; the integration between the two devices is even greater in *Highlighting text*, as the target and alternative values transmitted as part of a text are also the receptors for the response indicating the chosen value. On other occasions, the reception device appears well separated from the transmitter, on another screen or in another window, or on a separate answer sheet, whether simultaneous or not. Instructions have the same possible characteristics as the other devices.

### Physical Medium

**FIGURE 2:**
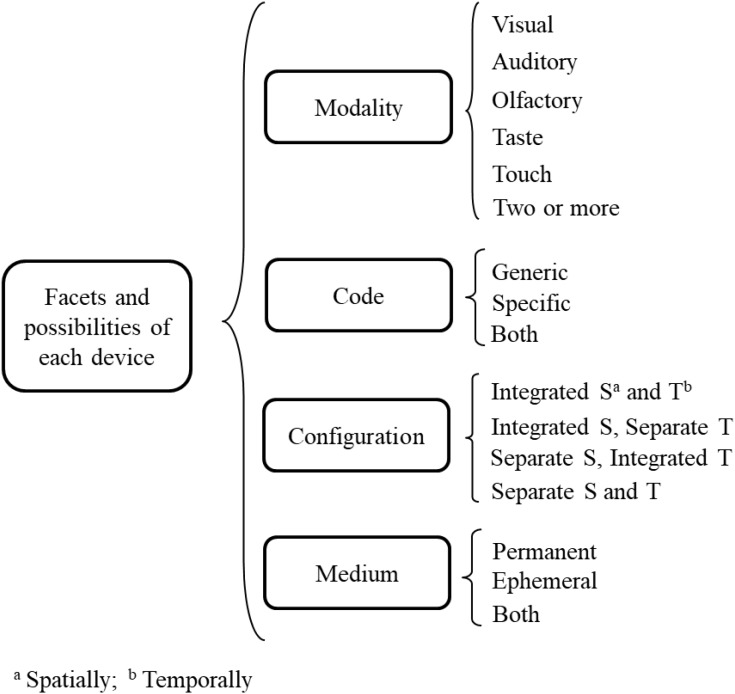
Possibilities of the facets for the transmission, reception, and instruction devices.

This device facet is the base material on which the items are presented. Frequently it is paper or a computer-, tablet-, or mobile-phone- screen for transmission and reception of sound and images. Other media include places, objects, or anything which can stimulate any of the sensory channels. It may include the evaluators themselves, giving instructions, or asking questions orally, or via sign language, and receiving the corresponding responses. All of the possible media may be classified as *permanent* if the media is stable and can preserve the device, such as computers, voice recorders, and physical objects such as paper or canvas. The alternative classification is *ephemeral* or transient, such as during an oral interview which is not recorded. As with all of the other facets, each mechanism in an item may include a combination of two or more media, such as a paper receptor to note answers to oral questions that are not recorded. Similarly, different devices in the same item may use different media, in any combination; for example, an oral item with a transmitter using an ephemeral medium, the air through which the sound propagates, with a recording as the permanent medium of the receptor. **Figure 2** summarises the possibilities of each device facet.

## Application of the Model

**FIGURE 3:**
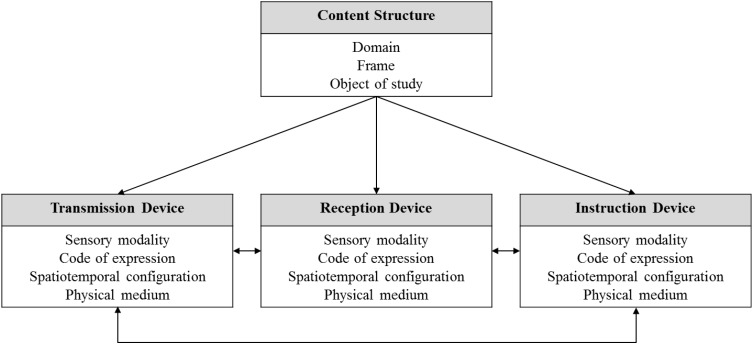
Components and facets of the proposed classification model.

As described above, item formats can be described in terms of the possibilities of the facets and components summarised in **Figure 3**. We illustrate the proposed model by demonstrating below how three specific items would be classified using the specifics of each item to illustrate the corresponding content structure.

The first item is number 1 in Beck’s Depression Inventory, version II (Beck et al., 1996). The facets of its content structure are: domain, psychological evaluation of people aged 13 or over; frame, the previous 2 weeks including the day of application; object of study, sadness felt; four values, ordered by level; the target value is the value chosen by the examinee, as in evaluations of attitude or personality, with the other three values being alternatives. The transmission device is the naming of the object of study and the four values. When it is applied in written form, whether self-administered or not, the facets of the transmission device are: Modality, visual; Code, generic from the language used; configuration, integrated in space and time as it is presented as a unit; and medium, permanent either paper or screen. The reception device consists of the numbers 0–3, assigned to the values of the transmitted object of study. The modality of the reception device is visual; the code is generic in the language used, and specific in what the item has of particular; the configuration is integrated in space and time; and the medium is permanent. The instructions are common for all of the items in the questionnaire. They explain the transmission device, indicating that each item is made up of four statements referred to the previous 2 weeks, and that the task for the examinees is to choose the statement they feel is most appropriate. The instructions also indicate how to express the answer in the reception device, asking the examinee to ensure that they have not selected more than one answer. The modality of the information device is visual, the code is generic in the language used, the configuration is integrated in space and time (as it is presented in a single unit), although spatially separated from the other two devices as it is provided before the items, and the media is permanent. If the item is applied orally, some of the facets will differ. The transmission device will be oral, presented via an ephemeral medium (being a non-recorded voice). The same will apply to the instructions, however, the reception device will be visual and oral, as the test administrator uses the inventory to collect the responses made by the examinee.

Many items in evaluations of attitudes and personality share similar classifications to the above, with some slight differences. One example is the items in the Minnesota Multiphasic Personality Inventory-2 (Butcher et al., 1989). Apart from the specific objects of study and frames for each item, such as satisfaction and one’s sex life (respectively) in item 12 “My sex life is satisfactory,” the facets of the transmission device are similar to the BDI in its written administration, with the exception that it only includes one value of the object of study, which the examinee converts into a target or alternative with their response, as it evaluates personality. The reception device is made up of two circles where the examinee records their response according to whether they think the transmitted content of the item is true or false. Its configuration is separated in space and time as it appears with the transmission device and also in a separate sheet of paper where the response is recorded. The instructions are also visual, they appear as an integrated unit, although separated from each item as they are common to all items. They describe the other two devices, indicating how to mark responses, giving the examinee the option of leaving an item response blank if they cannot choose between the two, and asking the examinees to take care that each answer corresponds to the appropriate item.

The items in the well known Raven’s Progressive Matrices Test-Standard (Raven et al., 1996) have similarities to the examples given above, but are particularly different in the transmission device. The transmission device in each item presents a frame of a rectangle containing black and white geometrical shapes drawn in black and white, missing one pattern in the bottom right corner, which is the object of study; below the rectangle, as possible values of the object of study, there are six similarly shaped images to the missing piece, each with a different drawing. Only one of those six is the target value which should go in the space, regardless of the examinee’s answer, as it is an evaluation of aptitude or competencies. The transmission device has the following facets: mode, visual; code, specific drawing for each item; configuration, integrated in space and time; and medium, permanent, whether on paper or on screen, as with all of the examples up to now. The reception device is made up of six circles presented in two rows of three, with numbers corresponding to each value of the object of study, in which to mark the chosen answer. The modality is visual, the code specific, the configuration is separated spatially from the transmission device, on a separate answer sheet. The instruction facets are similar to those described above in their written application.

Another item for illustration is item number 1 in the VII Facial-Nerve Item Test (The Neurological Exam, 2001). This consists of an applicator with a cotton tip impregnated with a solution which the examiner uses to lightly touch one side of the front of the examinee’s tongue, which they hold outside of their mouth. With the tongue still outside the mouth, the examinee is shown a piece of paper containing the names of four tastes, and asked to indicate which one was applied. The reason for this is if the examinee were allowed to put their tongue back in their mouth to verbalise their response, they could transfer the stimulus to the other side of their tongue, making the evaluation of possible lesions in the VII facial nerve more difficult. The item is analysed using an open checklist which facilitates the use of the proposed classification model (see **Table 1**).

**Table 1:** Classification of item 1 from the VII Facial-Nerve Test (The Neurological Exam, 2001).

Components and facets		Classification and description	
Content structure			
	Number of domains:	One	*Neurological evaluation*
	Number of frames:	One	*Tongue*
	Object of study:	One	*Taste*
	Number of targets:	One	*Salty*
	Number of alternatives:	More than one	*Bitter, Sour, and Sweet*
Transmission device			
	Modality:	Taste	*Taste*
	Code:	Generic	*Everyday tastes and Oral English*
	Configuration:	Integrated S^a^ and T^b^	*Application of a substance and a question at the same time*
	Medium:	Ephemeral	*A solution on a cotton tip touching the tongue, and transmission of sounds through the air*
Reception device			
	Modality:	Visual and tactile	*Names of target values written down for the examinee to indicate one*
	Code:	Generic	*Written English*
	Configuration:	Integrated S and T	*A table with the names of four tastes presented simultaneously*
	Medium:	Permanent	*A sheet of paper*
Instructions			
	Modality:	Oral	*Explanation of the reception device and how to use it to answer*
	Code:	Generic	*Oral English*
	Configuration:	Integrated S, and separated T	*Expression presented as a single unit, before the other two devices*
	Medium:	Ephemeral	*Non-recorded expression*


*^*a*^Spatially;^*b*^Temporally.*

The final illustration of the proposed system describes an information and communication technology (ICT) skill test item used as material in an experiment (Engelhardt et al., 2016, p. 686). An examinee is presented with an e-mail programme on a computer screen, the task to be completed appears in a column on the left. The examinee is asked to imagine that they work in a company which a new employee has joined who is not yet on the email distribution list. They are asked to forward the applicable emails to the new employee. The assessment is whether one of the emails in the examinee’s inbox is correctly identified as an email that should not be forwarded (see **Table 2**).

**Table 2:** Classification of an ICT skill test item (Engelhardt et al., 2016, p. 686).

Components and facets		Classification and description	
Content structure			
	Number of domains:	One	*ITC skills*
	Number of frames:	One	*Open email software*
	Object of study:	One	*Identification of an email that should not be forwarded*
	Number of targets:	One	*The email which should not be forwarded in the email inbox*
	Number of alternatives:	More than one	*Other emails in the inbox. Other folders if they are not already open in the email inbox (none open to the examinee -not mentioned in the example in the main text)*
Transmission device			
	Modality:	Visual	*Email software*
	Code:	Generic	*Parts of the software, each with its own meaning, and written English*
	Configuration:	Integrated S^a^ and separated T^b^	*Successive screens that the examinee needs to open, each one as an integrated unit*
	Medium:	Permanent	*Computer screen*
Reception device			
	Modality:	Visual and tactile	*The email software, accessed through the mouse*
	Code:	Generic	*Parts of the software, each with its own meaning; use of the mouse; and written English*
	Configuration:	Integrated S and separated T	*Successive screens that the examinee needs to open, each one as an integr ated unit*
	Medium:	Permanent	*Computer screen and mouse*
Instructions			
	Modality:	Visual	*Explanation of the task to the examinee.*
	Code:	Generic	*Written English*
	Configuration:	Integrated S and T	*Presented as a unit, together with the transmission device*
	Medium:	Permanent	*Computer screen*


*^*a*^Spatially;^*b*^Temporally.*

## Discussion and Conclusion

The review of test item format classification systems illustrates some of their limitations, such as having ambiguous classification criteria, insufficient differentiation, and referring almost exclusively to pen and paper or screen-based items and ignoring other formats. This study proposes a new taxonomic model as an alternative to the downsides of those taxonomies. The new model has four components (content structure, transmission device, reception device, and instruction device), and seven facets; three in the content structure component (domain, frame, and object of study), and four in the devices (modality, code, configuration, and medium). This model avoids the ambiguity and inconsistency of functional and morphological perspectives of item components. It uses both perspectives, albeit differently, with the morphological perspective being the necessary material embodiment of the functional perspective. In that way, the components and their facets are based on their function in the item: content structure is the reference for the devices, the transmission device communicates part of that structure and poses the question about it, the reception device collects the expression of the answer, the instruction device facilitates that, while the facets are how and where the four components are rendered. The devices’ facets in turn are identifiable in each item by their morphological characteristics, specified in the different possibilities of sensorial modality, code of expression, spatiotemporal configuration, and physical medium. The conjunction of functional and morphological perspectives should correspond to and serve the information to be transmitted and collected; so for example, memory tests should correspond to a configuration of devices which are separated in time between memorisation and recall of information.

Some components and facets of the taxonomy are similar to elements of other organising proposals for item development. One of these proposals is the framework described by Kirsch (2001) in six phases, one of which is the delimitation of a particular domain of interest, just like in the taxonomy proposed. The definition of the facets object of study, with their target and alternative values, and frames can be identified in the *Context/Content* section specification in the *Identifying and Operationalizing the Variables* phase of the Kirsch framework. Likewise, the constitutive of some facets of the format of the transmission and reception devices appear in the sections *Materials/Texts and Processes/Strategies*. Thus, the integrated and separated configurations considered in the taxonomy correspond to the distinction between *Continuous and Non-continuous Texts*, appearing in both types of texts several possibilities of the code of the devices, and of the response of the examinee in the reception device. Another proposal with similarities is the *Evidence Centred Approach* (Mislevy et al., 2003), designed for the entire process of developing and using tests, organised into five groups of activities called “layers.” Specifically, the content structure component of the taxonomy shares similarities with the *Domain Modelling Layer*, in which aspects of a domain of interest are selected to build an evaluation subject. In addition, the transmission and reception devices would fit into the element of the *Assessment Implementation Layer*, closely related to traditional test development jobs of writing items. In short, on the basis of these similarities, the use of the proposed taxonomy could be incorporated into the two frameworks indicated, as an aid in the task of deciding the format of each item to be developed.

In the new classification, the consideration of stems and options as item components (e.g., Osterlind, 1998; Downing, 2006; Haladyna and Rodriguez, 2013) is unnecessary because, in addition to being ambiguous and inconsistent, they do not allow as thorough a differentiation of the variety of formats as the possible characteristics presented above. For a single object of study, thousands of different item formats are possible from the combinations of the five possible modality facets, the four configuration facets, the two code facets, and the medium facet in each of the three devices, more so if one considers the combination of more than one in each device, and the combinations between the three devices, plus the possible facets of content structure that may be communicated in the transmission device. In addition, and as the majority of these formats have not yet been developed, the proposed classification is applicable both to existing and yet-to-be developed formats. In other words, this model has empty boxes (item types) ready for future development, which gives the model something of a generative nature, rather than being merely classificatory. It can serve as a guide for the construction of items with novel formats to a greater or lesser extent, something which was not within the objective of this study. For this purpose, the checklist offered in **Tables 1, 2** can be used considering the possibilities of the different facets. Also, to the extent that the model implies universal criteria of the formats, it can help in generating items which are invariant between groups and cultures. For this purpose, the model offers possible functional causes of the non-invariance of the measures, such as the facets of the physical medium or spatio-temporal configuration in the adaptations of paper and pencil formats to computerised tests (e.g., Vecchione et al., 2012; Vleeschouwer et al., 2014).

Apart from the replacement of stems and options as item components, other aspects of format noted in the literature have been preserved, albeit with some reinterpretation in the new classification. The multiple character of formats such as *Matching* and *Cloze* (e.g., Scalise and Gifford, 2006) is considered as a possible characteristic of the *Number of target values* in the object of study and the *Number of frames* in the transmission device. The number of options, indicated by format labels such as *Alternative Choice*, *Three-Option*, and others (Haladyna and Rodriguez, 2013), is preserved as it corresponds functionally with the *Number of target values and alternatives*. The distinction between selected and constructed-response formats is seen as possible responses requested as a function of responses transmitted, although without the central role it usually plays. Similarly, the defining features of formats such as *Grid-in*, *Graphical Modelling* (e.g., Sireci and Zenisky, 2006) and many others are covered by devices’ *Codes*.

The components and facets proposed here facilitate the evaluation of whether the format of each item helps it fulfil its function. This will allow the valid collection of a response as long as it is expressed in the reception device, following the instructions given which fits the content structure proposed in the transmission device. To that end, the possibilities used in each facet and component must be specified with sufficient precision, and differentiated from undesired possibilities. Imprecision in the components may oblige the examinee to interpret them, with the consequent risk of not doing what the item wants, and losing validity of the information so collected. In this sense, the classification proposed here may also promote and organise research on the influence of different format possibilities on item validity.

## Author Contributions

RM, RJM, and JM contributed in a similar way to the interactive process followed for the construction and presentation of the proposed taxonomy. The only difference is that RM wrote the first version and the subsequent modifications.

## Conflict of Interest Statement

The authors declare that the research was conducted in the absence of any commercial or financial relationships that could be construed as a potential conflict of interest.

## References

[B1] Anderson J. A. (1996). *Communication Theory: Epistemological Foundations.* New York, NY: The Guilford Press.

[B2] Beck A. T. Steer R. A. Brown G. K. (1996). *Beck Depression Inventory* 2nd Edn. San Antonio, TX: Psychological Corporation.

[B3] Byrne B. M. van de Vijver F. J. R. (2017). The maximum likelihood alignment approach to testing for approximate measurement invariance: a paradigmatic cross-cultural application. *Psicothema* 29 539–551. 10.7334/psicothema2017.178 29048316

[B4] Butcher J. N. Dahlstrom W. G. Graham J. R. Tellegen A. Kaemmer B. (1989). *The Minnesota Multiphasic Personality Inventory-2 (MMPI-2): Manual for Administration and Scoring.* Minneapolis, MN: University of Minnesota Press.

[B5] Downing S. M. (2006). “Selected-response item formats in test development,” in *Handbook of Test Development* eds Downing S. M. Haladyna T. M. (Mahwah, NJ: Lawrence Erlbaum Associates) 287–302.

[B6] Drasgow F. (2016). *Technology and Testing.* New York, NY: Routledge.

[B7] Engelhardt L. Goldhammer F. Naumann J. Frey A. (2016). Experimental validation strategies for heterogeneous computer-based assessment ítems. *Comp. Hum. Behav.* 76 683–692. 10.1016/j.chb.2017.02.020

[B8] Gallager R. (1968). *Information Theory and Reliable Communication.* New York, NY: John Wiley and Sons.

[B9] Gómez-Benito J. Balluerka N. González A. Widaman K. F. Padilla J. L. (2017). Detecting differential item functioning in behavioral indicators across parallel forms. *Psicothema* 29 91–95. 10.7334/psicothema2017.183 28126065

[B10] Haladyna T. M. Rodriguez M. C. (2013). *Developing and Validating Test Items.* London: Routledge.

[B11] Helbig H. (2006). *Knowledge Representation and the Semantics of Natural Language.* Berlin: Springer-Verlag.

[B12] International Test Commission (2017). *The ITC Guidelines for Translating and Adapting Tests* Available at: www.InTestCom.org

[B13] Kirsch I. (2001). *The International Adult Literacy Survey (IALS): Understanding What Was Measured (Research Report 01-25).* Princeton, NJ: Educational Testing Service.

[B14] Magno C. (2009). Taxonomy of aptitude test items: a guide for item writers. *Int. J. Educ. Psychol. Assess* 2 39–53.

[B15] Mislevy R. J. Almond R. G. Lukas J. F. (2003). *A Brief Introduction to Evidence-Centered Design (Research Report 03-16).* Princeton, NJ: Educational Testing Service.

[B16] Moreno R. Martínez R. Y. Muñiz J. (2015). Guidelines based on validity criteria for the development of multiple choice items. *Psicothema* 27 388–394. 10.7334/psicothema2015.110 26493578

[B17] Muñiz J. Elosua P. Hambleton R. K. (2013). Directrices para la traducción y adaptación de los tests: segunda edición [Guidelines for test translation and adaptation: Second edition]. *Psicothema* 25 151–157. 23628527 10.7334/psicothema2013.24

[B18] Osterlind S. J. (1998). *Constructing Test Items: Multiple-Choice, Constructed-Response, Performance and Others Formats.* Boston, MA: Kluwer Academic Publishers.

[B19] Osterlind S. J. Merz W. R. (1994). Building a taxonomy for constructed-response test items. *Educ. Assess* 2 133–147. 10.1207/s15326977ea0202_2

[B20] Rauthmann J. (2011). Not only item content but also item formats is important: taxonomizing item format approaches. *Soc. Behav. Pers.* 39 119–128. 10.2224/sbp.2011.39.1.119

[B21] Raven J. C. Court J. H. Raven J. (1996). *Raven Manual: Section 3 Standard Progressive Matrices.* Oxford: Oxford Psychologists Press.

[B22] Rodriguez M. C. (2016). “Selected response item development,” in *Handbook of Test Development* eds Lane S. Haladyna M. R. Raymond T. M. (New York, NY: Routledge), 259–273.

[B23] Scalise K. Gifford B. (2006). Computer-based assessment in e-learning: a framework for constructing “intermediate constraint” questions and tasks for technology platforms. *J. Tech. Learn. Assess.* 4 Available at: http://www.jtla.org

[B24] Shannon C. E. (1948). A mathematical theory of communication. *Bell Syst. Tech. J.* 27 379–423. 10.1002/j.1538-7305.1948.tb01338.x

[B25] Sireci S. G. Zenisky A. L. (2006). “Innovative item formats in computer-based testing: in pursuit of improved construct representation,” in *Handbook of Test Development* eds Downing S. M. Haladyna T.M. (Mahwah, NJ: Lawrence Erlbaum Associates) 329–347.

[B26] Sireci S. G. Zenisky A. L. (2016). “Computerized innovative item formats,” in *Handbook of Test Development* eds Lane S. Raymond M. R. Haladyna T. M. (New York, NY: Routledge) 315–334.

[B27] The Neurological Exam (2001). *VII Facial Nerve – Taste. [Video File].* Available at: http://neuroexam.med.utoronto.ca/cranial_7a.htm

[B28] Vecchione M. Alessandri G. Barbaranelli C. (2012). Paper-and-pencil and web-based testing: the measurement invariance of the big five personality tests in applied settings. *Assessment* 19 243–246. 10.1177/1073191111419091 21862530

[B29] Vleeschouwer M. Schubart C. D. Henquet C. Myin-Germeys I. van Gastel W. A. Hillegers M. H. (2014). Does assessment type matter? A measurement invariance analysis of online and paper and pencil assessment of the community assessment of psychic experiences (cape). *PLoS One* 9:e84011. 10.1371/journal.pone.0084011 24465389 PMC3898946

